# The plant microbiota signature of the Anthropocene as a challenge for microbiome research

**DOI:** 10.1186/s40168-021-01224-5

**Published:** 2022-03-26

**Authors:** Gabriele Berg, Tomislav Cernava

**Affiliations:** 1 Institute of Environmental Biotechnology, Graz University of Technology, Petersgasse 12, 8010 Graz, Austria; 2 Leibniz-Institute for Agricultural Engineering Potsdam, Max-Eyth-Allee 100, 14469 Potsdam, Germany; 3 Institute for Biochemistry and Biology, University of Potsdam, Karl-Liebknecht-Str. 24/25, 14476 Potsdam, Germany

## Abstract

**Background:**

One promise of the recently presented microbiome definition suggested that, in combination with unifying concepts and standards, microbiome research could be important for solving new challenges associated with anthropogenic-driven changes in various microbiota. With this commentary we want to further elaborate this suggestion, because we noticed specific signatures in microbiota affected by the Anthropocene.

**Results:**

Here, we discuss this based on a review of available literature and our own research targeting exemplarily the plant microbiome. It is not only crucial for plants themselves but also linked to planetary health. We suggest that different human activities are commonly linked to a shift of diversity and evenness of the plant microbiota, which is also characterized by a decrease of host specificity, and an increase of r-strategic microbes, pathogens, and hypermutators. The resistome, anchored in the microbiome, follows this shift by an increase of specific antimicrobial resistance (AMR) mechanisms as well as an increase of plasmid-associated resistance genes. This typical microbiome signature of the Anthropocene is often associated with dysbiosis and loss of resilience, and leads to frequent pathogen outbreaks. Although several of these observations are already confirmed by meta-studies, this issue requires more attention in upcoming microbiome studies.

**Conclusions:**

Our commentary aims to inspire holistic studies for the development of solutions to restore and save microbial diversity for ecosystem functioning as well as the closely connected planetary health.

Video abstract

**Supplementary Information:**

The online version contains supplementary material available at 10.1186/s40168-021-01224-5.

## Introduction

Anthropogenic activities have shaped our planet in such a drastic way that they have resulted in the definition of a human-dominated geological epoch, the Anthropocene [1]. There is a direct link between continuous human population growth, overpopulation of certain areas, overconsumption and intense agriculture; together they constitute major drivers of the Anthropocene [1]. The Anthropocene is also reflected in the planetary boundary concept; four of the nine boundaries have already been crossed: extinction rate, deforestation, climate change, and the flux of nitrogen and phosphorus [2]. However, little is known about the impact of these anthropogenic factors on different host-associated and environmental as well as inter-linked microbiomes and their consequences for our planet [3]. Studies on the human microbiome indicated a substantial biodiversity loss connected with an increase of chronic diseases [4, 5]. The discovery of this correlation marks a fundamental breakthrough for the recognition of the importance of microbial biodiversity but it needs a much deeper understanding about driving factors, modes of action and consequences. Furthermore, the question arises as to how specific this loss of biodiversity is and if some populations are more affected than others. This observed diversity loss needs also a better inter-linking with the environmental microbiome and exposome; the plant microbiome is an interesting example for both [6]. Although the impact of human activity on the plant-associated microbiota can be studied with various experimental approaches [7], it is still little understood and needs more attention in terms of global food security and safety but also in frame of inter-linked microbiomes and planetary health [2, 3, 8].

More than a century ago, Lorenz Hiltner discovered the importance of plant-associated microorganisms for plant growth and health [9]. In the last 20 years, technology-driven microbiome research substantially contributed to better insights into host-microbiota relationships [6, 10]. Now, each plant is considered as a meta-organism or holobiont consisting of a plant host and billions of specific microbial cells with a certain dependency on metabolite exchange [11]. The microbiota is involved in the host plant’s germination, growth, productivity, adaptation, physiology, health and has contributed to diversification within the plant kingdom [11, 12]. This symbiotic functional interplay can be explained by plant-microbe co-evolution, and the plant genotype was shown to be one of the most important drivers [13]. The composition of the plant microbiota varies during a plant’s life cycle and is vertically transmitted and horizontally influenced [14]. While environmental drivers are well studied [15, 16], the impact of anthropogenic factors on the microbiome—the main drivers of global ecosystem changes nowadays—has received less attention; especially a holistic view on functional implications is missing.

During the last years, which were characterized by an enormous knowledge increase related to the plant microbiome, we noticed a common microbiome shift in the Anthropocene. Therefore, we want to further elaborate this in the context of our microbiome definition which also presented concepts for unifying concepts and standards [6]. This is important to stimulate more attention, research, and solutions on this topic. While the plant microbiome is used as an example, in the overall context of microbiome exchange, it is important to consider that the plant microbiome is inter-connected with other microbiomes, e.g., those in the soil, animals, and humans; however, the detailed exchange processes between the different interfaces are still little understood [6]. It is known that such interfaces can be crossed by antimicrobial resistance (AMR) determinants as well, which are anchored in the microbiome [17]. In this article, we briefly summarize current knowledge related to anthropogenic influences on the plant microbiome and discuss the following hypotheses: (i) a substantial proportion of plant-specific microbiota could get extinct along with their hosts, (ii) anthropogenic impact has globally induced significant microbiome shifts and, (iii) the Anthropocene results in unique adaptations of resident microbial communities, resulting in novel assemblages, activities, and features that differ from those in natural environments. The plant microbiome is strongly connected with *One* Health and planetary health issues [18]. Therefore, the main aim is to inspire the development solutions to restore and save plant-associated microbial diversity to maintain ecosystem functioning which is closely connected to human health.

## Results

### Loss of plant biodiversity is accompanied by macrobiotic extinction

Plants provide the fundamental basis for ecosystem functioning and food chains in nearly all terrestrial ecosystems on Earth. All plants are considered as holobionts; therefore, plant-associated microbial diversity is strongly connected with the biodiversity of plants [19]. Declines in plant species diversity and extinctions in the Anthropocene are associated with habitat conversion, climate warming, habitat fragmentation, and nitrogen deposition mainly for intense agricultural production. Recently, in the frame of the largest global survey, botanists have reported alarming extinction rates for native plants; up to 500 times higher than would be expected as a result of natural forces alone [20]. Plants on islands and in the tropics were the ones most likely to be declared extinct, while trees, shrubs, and other woody perennials had the highest probability of disappearing regardless of their location. Among the many threatened species are wild relatives of our main crop plants—the wild and weedy cousins of domesticated plants that possess valuable traits for crop breeding, such as pest and disease resistance. The displacement of these plants is favoured by human nutrition, mostly because diets across the world have become more homogeneous and are mostly based on a few staple crops. This has resulted in a decline in consumption of local or regionally important crops by two thirds [21]. The loss of plant and crop diversity has likely resulted in a loss of the associated, plant-specific microbiota; however, the quantification of the extent is currently difficult due to methodological problems and the limited coverage of plant species by microbiome analyses.

The plant microbiota generally consists of different ecological types, e.g., cosmopolitans and specialists; the proportion of specialists is known to vary between genotypes, species and phyla, and often proves beneficial, such as the mycorrhiza [6, 9, 13]. The theory that arbuscular mycorrhizal fungi (AMF) symbionts were drivers of plant terrestrialization in early Palaeozoic land ecosystems is well established but less is known about interactions with bacteria during plant evolution [13]. Non-vascular plants such as liverworts, hornworts and mosses (bryophytes) belonged to the first land colonizers. The low extant bryophyte species richness today is due to low diversification rates and massive extinction events, and especially many hornworts are still endangered from extinction. Recently it was discovered that they harbour a high, vertically transmitted microbial diversity and do not filter microorganisms out of the local environment as efficiently as vascular plants [22]. We can expect that specific AMF and other bryophyte-associated symbionts got lost in that long period; however, this topic needs further exploration. Loss of plant diversity is not only followed by the direct loss of specifically adapted microorganisms that used to be part of the now-extinct holobionts, but also has substantial implications for below ground diversity. The below ground diversity is regulated by biotic factors, but also responds to physicochemical changes that reinforce the overall effect. In conclusion, there is strong support for our first hypothesis: together with their host plants, a considerable part of the plant microbiota could also be threatened with extinction. More studies on the native/endemic and tropical vegetation are required to avoid that a substantial part of the plant microbiota gets extinct before their discovery. However, the impressive diversity of plants reflected in our vegetation today, as a result of long-term plant-microbiome co-evolution [11], supports our third hypothesis as well. Under anthropogenic pressure, unique adaptations can result in novel assemblages, activities, and features.

### Signatures of the plant microbiome in the Anthropocene

Several key features such as climate change, changing biogeochemical cycles, and pollution characterize the Anthropocene [2]. The impact of many of them on the plant microbiome is already studied and partially mechanistically understood, but there is no complete picture of the Anthropocene’s impact yet. Due to the high dynamics of change and multifactorial impacts, this picture is difficult to draw. Interestingly, Rillig et al. [7] provided experimental evidence that increasing the number of simultaneous global change factors caused increasing directional changes in soil microbial communities, though there was greater uncertainty in predicting the magnitude of change. Here, we try to link the impact to single and selected combined parameters, and draw conclusions related to general qualitative tendencies of Anthropocene-induced microbiome shifts. A representative selection of anthropogenic effects on the plant microbiome, based on evidence in the form of experimental and metadata studies, is described in detail and listed in Table 1.

**Table 1:** Examples for the anthropogenic impact on microbiome signatures in plant holobionts and in terrestrial ecosystems in the Anthropocene from all over the world

Anthropocene signature	Analyzed factor	Ecosystem/holobiont	Resulting microbiome signatures	Reference
**Climate change**	Global warming	Cropping systems	Warmer temperatures cause an increase of the relative abundance of soil-borne fungal plant pathogens.	[23]
		Cherry	Warming increased the abundance of fungal plant pathogens with higher host infection rates as a consequence.	[24]
		Bog ecosystem	Microbiome shifts were observed in controlled warming experiments. A decreased diversity of bacteria and diazotrophs as well as a reduced nitrogen fixation rate was observed.	[25]
		Oak trees	Increased temperature resulted in lower microbial diversity under controlled conditions. It was also followed by an increase in pathogen occurrence.	[26]
		Grasslands	A decreased ‘drift’ was observed over time, which enhances homogeneous selection that is primarily imposed on *Bacillales.*	[27]
		Soil leaf litter layer	A short-term adaptation and altered diversity were observed. Non-random, parallel mutations in genes related to nutrient acquisition, stress response, and exopolysaccharide production were characteristic for adaption.	[28]
	Drought	Grasslands	Changes in soil functioning and plant community composition were observed and shown to be shaped via the modification of plant–soil feedbacks under drought conditions.	[29]
		Pine and oak trees	Microbiota shifts and a decrease in diversity were reported.	[30]
	Erosion	Soil	Adaptions were characterized by low microbial network complexity. A decrease in functionality but increase in the relative abundances of some bacterial families involved in N cycling, such as *Acetobacteraceae* and *Beijerinckiaceae* was observed.	[31]
**Nitrogen and phosphorus flow disturbances**	Nitrogen fertilization	Wheat roots and rhizosphere	Overuse of nitrogen fertilizers causes microbiome shifts towards Proteobacteria.	[32]
		Wheat rhizosphere	Bacterial community richness and diversity decreased after plants were supplemented with inorganic nitrogen.	[33]
		Soil	Protist diversity is indirectly reduced by bacterial and fungal community shifts caused by nitrogen inputs in agricultural soils	[34]
		Different forest ecosystems	Nitrogen fertilization substantially reduced the diversity and abundance of nitrogen-fixing bacterial communities under elevated atmospheric CO_2_ conditions.	[35]
	Phosphorous fertilization	Soil (ryegrass)	One-time inorganic phosphate amendments caused shifts in soil bacterial and fungal communities and reduced mycorrhization rate in ryegrass.	[36]
	Phosphorous and nitrogen fertilization	Barley	Long-term nitrogen fertilization was shown to affect arbuscular mycorrhizal fungal communities while long-term phosphorous fertilization limited phosphorous provision to plants.	[37]
**Chemical pollution**	Microplastics	Soil	Contamination of different soils with microplastics resulted in a specific enrichment of antibiotic resistance genes. The effect was further enhanced by elevated temperature.	[38]
	Antibiotics, heavy metals, and microplastics	Soil	Enhanced antibiotic resistance occurrence was observed in manured soil.	[39]
	Microplastics	Soil	Altered soil and microbiome structure were liked to microplastics contamination.	[40]
	Neonicotinoid seed treatments	Phyllosphere and soil in soybean-corn agroecosystem	Microbiota shifts were reflected by a decline in the relative abundance of some potentially beneficial soil bacteria (bacteria involved in the N cycle) in response to pesticide applications.	[41]
	Engineered nanomaterials: SiO_2_, TiO_2_, and Fe_3_O4	Maize rhizosphere	A reduction of N-fixing bacteria and iron-redox bacteria was reported along microbiome shifts. Occurrence of plant growth promoting bacteria was enhanced.	[42]
	Broad-spectrum fungicide: N-(3,5-dichlorophenyl) succinimide	Tobacco phyllosphere	Pesticide applications caused a microbiome shift towards a higher prevalence of Gammaproteobacteria in the phyllosphere of treated plants.	[43]
	Antibiotic treatment	Oilseed rape	Mutation frequencies can explain differentiation between plant and clinical *Stenotrophomonas maltophilia* strains. Clinical environments might select bacterial populations with high mutation frequencies.	[44]
**Biodiversity loss**	Breeding of high-yield crops	Various crop plants	An overall tendency of microbiome shifts from k- to r-strategists was demonstrated.	[45]
	Breeding of high-yield crops	Maize	It was shown that more recently developed germplasm recruited fewer microbial taxa with the genetic capability for sustainable N provisioning and larger populations of microorganisms that contribute to N losses.	[46]
**Stratospheric ozone depletion**	UV-B radiation	Peanut phyllopshere	Characterization of 200 phyllosphere isolates indicated that the predominant UV-tolerant members were *Bacillus coagulans, Clavibacter michiganensis,* and *Curtobacterium flaccumfaciens.*	[47]
		Maize phyllosphere	UV-B radiation can affect bacterial diversity in the phyllosphere via the host plant’s gene products encoded on identified chromosomal quantitative trait loci (QTL).	[48]
		Maize phyllosphere	A strong tendency toward increased 16S rDNA sequence diversity was observed in UV-exposed samples.	[49]
**Combined effects**	Agricultural intensification	Various crop plants	A reduced network complexity and a reduced abundance of keystone taxa were described.	[8]
	Diverse	Global microbiome	An enrichment of Firmicutes and hypermutation genes in global microbiomes was observed.	[50]
	Diverse	Soil	Local increase of bacterial diversity and a global-scale homogenization of the soil microbiome was described. Additionally, soil-borne fungal pathogens were shown to accumulate which is accompanied by a reduction of beneficial microbes.	[51]
	Drought and nitrogen availability	Rhizosphere of *Alhagi sparsifolia*	Rhizospheric fungi are more sensitive to N and water addition than bacteria. Low N input and drought increased microbial co-occurrence network complexity.	[52]

Climate change has been identified as a core planetary boundary, which has the potential to drive global systems into a new state should it be substantially and persistently transgressed [2]. Climate change is expressed by several conditions, e.g., global warming, stratospheric ozone depletion or changing weather conditions. All of them were shown to have an impact on the plant microbiota (Table 1). In detail, Delgado-Baquerizo et al. [23] provided predictions for the introduction of new pathogens into production areas that were spared so far, especially fungal plant pathogens. Experimental evidence for the impact of global warming on pathogens was provided with *Prunus padua* plants, which showed a significantly increased abundance of pathogenic fungi and infections [24]. Bacterial and fungal populations often show negative co-occurrences within the plant microbiome. The introduction of non-native fungal species could lead to the depletion of certain microbiome members that are antagonized or compete with the intruders. In the frame of the SPRUCE macrocosm experiment, Carell et al. [25] deciphered that a microbiome shift in *Sphagnum* was connected with a decreased diversity of bacteria and diazotrophs as well as a reduced nitrogen fixation rate. Another warming experiment that was carried out under controlled conditions provided clear evidence that the diversity of oak-inhabiting fungi is reduced [26]. Microbiota shifts and fast selection processes were described in various studies, where the taxonomy of selected species showed variations, but also showed a clear tendency towards pathogens and spore-forming organisms [27]. Another factor, stratospheric ozone depletion results in increased levels of UV-B radiation, and adversely affects plant fitness; studies revealed decreases in plant height and shoot mass as well as a reduction in foliage area [53]. The foliage area constitutes a major part of the phyllosphere, which is one of the largest terrestrial ecosystems for microbial communities. The terrestrial leaf surface area is estimated to exceed 10^8^ km^2^ globally [54]. Due to the continuous reduction of this habitat and the impact of UV-B radiation on phyllosphere communities itself, a loss of the host plant’s native microbial diversity can be expected together with the formation of adapted communities, which have a higher resistance towards UV-B radiation. Various studies have provided clear evidence for the strong effects of UV-B radiation on the microbiota of various host plants [47–49]. Biogeochemical cycles, mainly driven by microorganisms, ensure functioning of ecosystems on our planet. The excessive use of nitrogen-containing fertilizers is particularly damaging, because a substantial amount of the nitrogen that is not taken up by plants is transformed into nitrate which is easily leached. Moreover, it was shown that N as well as P fertilizer usage resulted in loss of important functions for mycorrhiza and plant-associated bacteria, especially implications on diversity and negative regulation of distinct functional genes was often observed (Table 1). Nevertheless, it is important to highlight that the availability of chemical fertilizers has ensured nutrition of the human population by substantially enhancing crop yields in the first period of the Anthropocene. It was only later that it was realized that crop cultivars that were bred and treated for high yields, are not resilient plants, they are more susceptible to diseases than older cultivars. Due to these circumstances, larger quantities of pesticides are needed in agriculture nowadays. For example, many crop seeds are not able to grow anymore without chemical strippers, and the ripening process has to be supported by chemical treatments. In all these issues, a microbial dysbiosis can be involved, because all these are functions, which can be supported by the indigenous plant microbiome [6, 11]. Another measurable chemical signal of the Anthropocene are increased concentrations of specific pollutants that were released into the environment at the highest rates during the 1950s. These pollutants, encompassing inorganic and organic compounds have been widely studied in sediments and ice cores, and it was shown that they often have a high persistence potential and remain, even after being banned, long-term in the environment [55]. Especially the global spread of microplastics, and persistent organic pollutants was already shown to have a strong potential to shape plant-associated microbial communities as well as their antibiotic resistance gene (ARG) repertoire [38]. In a specific case, it was shown that the common presence of antibiotics, heavy metals, and microplastics can synergistically enhance antibiotic resistance in manured soil [39].

In summary, human activities affect the microbiome by altering colonization, exerting selection through our adapted and increasingly homogenous diet, and by changing species composition via use of various antimicrobials and pollutants. Microbial evolution has to keep pace with the environmental changes wrought by humanity. It remains to be seen whether organisms with long generation times, smaller populations and larger sizes are able to keep up. The accumulation of r-strategic bacteria, which are fast-growing generalists that also include many members of *Enterobacteriaceae*, in the plant microbiome is a general trend connected with plant breeding and intensive cultivation of perennial plants (Table 1). So far, there is no clear link between the increased occurrence of r-strategists and specific phenotypic plant traits of extensively bred cultivars; however, it is likely that (i) increased amounts of readily available carbohydrates, (ii) shorter growth periods and faster ripening, and (iii) a loose connection between plants and their indigenous microbiota synergistically facilitate the establishment of microbial communities dominated by r-strategists. This trend can be clearly observed with several crop plants that were subjected to extensive plant breeding [45, 56]. Their phenotypes were substantially changed within a relatively short period of time in order to increase the proportion of edible plant tissues. This has presumably led to a substantial accumulation of *Enterobacteriaceae* populations and other *Proteobacteria* in these plants, which are characterized by fast growth rates and thus well-suited to colonize modern cultivars of various unrelated plant lineages [57]. On the other hand, large-scale monocultures are connected to losses of biodiversity in soil due to the reduced chemical diversity, which naturally originates from root exudates. Monocultures can deplete plant-specific microorganisms from soils and thus negatively affect their role as reservoirs for microbial diversity. Banerjee et al. [8] demonstrated that agricultural intensification reduces network complexity and the abundance of keystone taxa in the root microbiome. In parallel, this was also shown to be a consequence of high-yield breeding [45, 57].

Altogether, recent literature indicates that losses and shifts of microbial diversity are a yet mostly overseen signature of the plant microbiome in the Anthropocene. This signature is often associated with changes in microbial diversity (abiotic factors often increase diversity; Table 1) and can result in dysbiosis, which leads to a higher susceptibility to plant pests and pathogens. Interestingly, applying the r/K selection theory, allows to predict an enrichment of fast growing microbial r-strategists while K-strategists will gradually disappear. R-strategist have a fast doubling rate, tolerance to more toxic compounds, and can thrive on a wide range of organic nutrients. Unlike r-strategists, K-strategists have lower growth rates and a better ability to exploit specific ecological niches. The latter are essential to stabilize ecosystems, fulfill unique functions, and they serve as a rich source of novel functions [58]. In addition, Song et al. [59] found time-related changes in various metagenomes, revealing a distinct r-related strategy with greater abundance of genes related to regulation and cell signaling, and a K strategy rich in motility and chemotaxis-related genes. Chen et al. [58] expanded this concept by including average 16S ribosomal RNA gene copy numbers, codon usage bias in ribosomal genes and predicted the maximum growth rate. The prevalent cultivation of annual crops instead of the natural perennial vegetation has resulted in the accumulation of r-strategic microorganisms, which are better adapted to the changing conditions. Another strategy for fast adaptations is the employment of hypermutation genes by various microbes; their enrichment was recently observed in selected taxa like *Firmicutes* and reflected specific ecological conditions and lifestyles for which hypermutation is advantageous [50]. In terms of adaptability to the Anthropocene, especially endophytic microorganisms that are intimately connected to their hosts and that have sacrificed a portion of their genetic repertoire in order to optimize their functioning [15] are likely more threatened by plant extinctions than their free-living relatives. The co-dependency of different organisms is often an effective strategy to survive under harsh conditions that would be otherwise restrictive for each of the contributing partners. Depletion of such organisms therefore also negatively affects their hosts and results in an increased dependency on agrochemicals.

On the one hand, declines in plant species diversity and extinctions are suggested as key signatures of the Anthropocene. However, on the other hand the introduction of non-native species has increased plant species richness in many regions of the world. This has led to the creation of new hybrid polyploid plant species by bringing previously isolated congeners into close contact [60]. Such processes affect microorganisms and microbiomes as well; selection pressure shapes evolution of microorganism and microbiomes in all plants. However, the extent to which microorganisms diversify and adapt to the changed to man-made conditions remain mostly unclear. Unique adaptations in resident microbial communities can result in novel assemblages, activities, and features that differ from those in natural environments. For example, novel hosts, high levels of nutrients, microplastics, and other pollutants can support horizontal gene transfer between microorganisms [39]. The expected consequences include plant-specific shifts of the microbiome as well as within the embedded resistome.

**Fig. 1:**
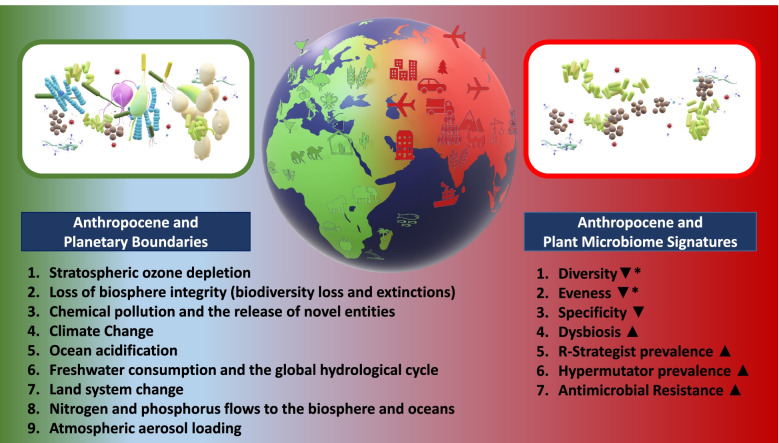
Planetary Boundaries and their impact on microbiome signatures in plant holobionts. *Increased evenness and diversity of plant microbiomes are often observed as a response to abiotic stress factors. In the long term, these changes can lead to dysbiosis and higher susceptibility to pathogens (biotic stress) which is commonly connected to decreased evenness and diversity

In conclusion, we suggest that a common signature of the plant microbiome in the Anthropocene is characterized by a decrease of specificity due to an increase of better adapted r-strategists, pathogens and hypermutators as well as specific antimicrobial resistance gene carriers (Fig. 1). Spatial and temporal scales and the overall extent of impacts are of great importance in the evaluation of microbiome studies [6]. In this context, it is known that intermediate disturbances can induce an increase in diversity and evenness, while strong and long-term disturbances induce the opposite. This is also reflected in the plant microbiome; it was commonly observed that the onset of abiotic stress can increase the diversity and evenness of microbial communities. Such conditions, especially warmer temperatures, often increase the prevalence of plant pathogens, which have a negative effect on diversity and evenness in the long term (Table 1). This confirms the applicability of the intermediate disturbance hypothesis for microbial ecology. Overall, it can be expected that Anthropocene-driven evolution of new microbial properties is accelerated. It will likely result in a global homogenization of the plant microbiome in analogy to described processes in the soil [51]. This conclusion supports our initial hypotheses that the anthropogenic impact has induced a significant microbiome shift and will lead to unique adaptations in resident microbial communities, resulting in novel assemblages, activities, and features that differ from those in natural environments.

### The plant microbiome embedded in the One Health and planetary health concepts: disease outbreaks and spread of antimicrobial resistances

The role of the microbiome for plant health is well-accepted, while the importance of inter-linked microbiomes, especially those associated with plants, for health issues is currently less in the focus of ongoing research. The connection of human, animal, and plant microbiomes is evident in the “*One* Health” concept of the World Health Organization (WHO) as well as in the “planetary health” concept that includes environmental health and its relation to human cultures and habits [18]. Bringing common signatures of the plant microbiome in the Anthropocene in relation with the inter-connected microbiome provides an interesting perspective; together the different components may result in intensified shifts, and possibly create a global microbiome imbalance.

If the balance in a microbiome is disturbed, outbreaks of pathogens occur where one microorganism can outgrow others. Whether such natural processes develop into diseases, epidemics or pandemics depends on many factors, including the virulence of the pathogen, i.e., its ability to infect, and the sensitivity of the host. Pathogens naturally occur in microbiomes of eukaryotic host. Each host-associated microbiome can contribute to disease formation with differing severity and the discovery of complex diseases has led to the introduction of the term pathobiome [61], which is characterized by a severe dysbiosis and often but not always connected with diversity loss. Change in diversity depends on the extent and duration of ecological disturbances; intermediate disturbances generally increase diversity while intense or prolonged disturbances decrease it. Deciphering the pathobiome will therefore become important for understanding persistence, transmission, and emergence of pathogens. During the SARS-CoV-2 pandemic it was observed that the implemented practices have substantially contributed to increased biodiversity loss and a corresponding loss of resilience [62]. Researchers even warn of further consequences of the pandemic for the microbiome and human health [63].

Antimicrobial resistances are recognised as a global societal challenge by the WHO. All plants are an important but mostly neglected reservoir for antimicrobial resistance (AMR) mechanisms [64]. Their microbiome harbours intrinsic antimicrobial resistances genes (ARGs), mobile genetic elements encoding resistance determinants and multi-resistant strains as well [65]. The resistome is embedded in the microbiome; thus the microbiota structure determines the resistome. Therefore, all drivers of the host plant’s microbiota, including genotype, secondary metabolites, microhabitats, and environmental factors, influence the composition of resistomes. In vascular plants and mosses that were studied so far, efflux pumps were shown to prevail in the ARG repertoire [65, 66]. Their mode of action is generally broad and unspecific, thus their function is most likely to provide resilience towards different biotic and abiotic stresses, and therefore of great importance for ecosystem functioning. We hypothesize that intrinsic AMRs provide a natural “health insurance” for the plant microbiome that is effective against harsh or changing environmental conditions and other external biotic and abiotic stresses. Considering that plant microbiomes are embedded in ecosystems and food cycles, plant resistomes must be considered in the *One* Health concept as well. Anthropogenic impacts trigger AMR formation and result in resistome shifts. The overall changes in the plant resistome are characterized by a shift from unspecific to specific AMR mechanisms, a decrease of AMR diversity and increase in AMR frequency within the microbiome as well as an increase of plasmid-associated ARGs. Native plants harbour a highly diverse pool of ARGs at low abundance, while anthropogenic influences reduce their diversity and enhance abundance. To avoid the spread of AMRs, the preservation of microbial biodiversity is crucial. In addition, Kim and Cha [17] suggested to improve the understanding and control of ARG transmission by (i) ranking the most critical ARGs and their hosts; (ii) understanding ARG transmission at the interfaces of *One* Health sectors; (iii) identifying selective pressures affecting the emergence, transmission, and evolution of ARGs; and (iv) elucidating the mechanisms that allow an organism to overcome taxonomic barriers in ARG transmission.

## Conclusions

Globalization, urbanization, overpopulation, and intensive agriculture together with other unsustainable resource utilizations have initiated the Anthropocene, the Age of Man. Biodiversity, which normally acts as a natural “health insurance” against pandemics, has declined drastically. A rethinking of our actions is urgently needed to bring our environment back into balance. Future studies, as well as reductions in Anthropocene-related impacts on the plant microbiome, could be stimulated if the following points are considered:

1. Several microbiota signatures of the Anthropocene are already confirmed by meta-studies. However, their in-depth understanding in terms of functional consequences requires more attention in upcoming microbiome studies.
2. The microbiota signature is important for all microbiome management approaches. We need holistic studies for the development of solutions to restore and save microbial diversity for ecosystem functioning as well as the closely connected planetary health.
3. Deciphering key stone species is important to better understand plant health and the connected *One* Health issues. Recent ecosystem and plant microbiome research has shown that certain microbes can be health determinants [67, 68]. We have to intensify the identification of beneficial key stone species (not only pathogens) within microbiome cycles, and follow their transmission routes, e.g., *Akkermansia*, *Lactobacillus*, and *Pseudomonas*.
4. Plant microbial diversity plays a key role for agricultural solutions. Intense agriculture is one of the major drivers of the negative facets of the Anthropocene. Changes towards sustainable agriculture are urgently required, e.g. more crop and cultivar diversity, reduction of fertilizers, pesticides and microplastics, introduction of crop rotation, agroforestry, intercropping, minimal tillage, and organic farming. The plant microbiome itself is a key feature for these solutions. It can be implemented in form of transplants, consortia and bioactive ingredients, which can be discovered by “microbiome mining”. Seeds (especially of wild relatives) and their specific microbiomes are of special importance for that.
5. Antimicrobial resistance is a double-edged sword. On the one hand, it ensures the plasticity of the plant microbiome and enhances resilience. On the other hand, it can be harmful for human health. More research focusing on human influences will be essential.
6. Rethinking plant sterility and microbial diversity is necessary. Tracking not only pathogens and AMRs, but also beneficial microbes within the plant microbiome should be considered for future risk assessments in crop production. This will ensure that untargeted damages with broad implications are avoided. This development is a societal challenge, which will require more education and translation for the society.
7. Our diet is crucial for future developments in agriculture and drives its impact on the Anthropocene. Eat fresh and local; eat more plants! There is an increased tendency around the globe to eat more plant-based food in order to reduce consumption of animal products. Taking into consideration that consumption of local products can reduce carbon footprints could help to further reduce our impact on climate. Fresh plants are healthier than processed ones and allow the transfer of their potentially beneficial microbiota to our guts.

## Data Availability

Not applicable.
